# Innovative Use of the Law to Address Complex Global Health Problems: Comment on "The Legal Strength of International Health Instruments - What It Brings to Global Health Governance?"

**DOI:** 10.15171/ijhpm.2017.62

**Published:** 2017-05-20

**Authors:** Helen L. Walls, Gorik Ooms

**Affiliations:** Faculty of Public Health and Policy, London School of Hygiene and Tropical Medicine, London, UK.

**Keywords:** Governance, Policy, Law, Global Health

## Abstract

Addressing the increasingly globalised determinants of many important problems affecting human health is a complex task requiring collective action. We suggest that part of the solution to addressing intractable global health issues indeed lies with the role of new legal instruments in the form of globally binding treaties, as described in the recent article of Nikogosian and Kickbusch. However, in addition to the use of international law to develop new treaties, another part of the solution may lie in innovative use of existing legal instruments. A 2015 court ruling in The Hague, which ordered the Dutch government to cut greenhouse gas emissions by at least 25% within five years, complements this perspective, suggesting a way forward for addressing global health problems that critically involves civil society and innovative use of existing domestic legal instruments.

The determinants of many important problems affecting human health are increasingly globalised, and addressing them is a complex task requiring collective action.^1^ Part of the solution likely lies with the role of international treaties, as described in the recent article of Nikogosian and Kickbusch.^2^ Law has been used to address tobacco control at both national and global levels – at global level most notably through the World Health Organization (WHO) Framework Convention on Tobacco Control (FCTC). However, as the authors describe, there is considerable scope for the use of law to address health issues beyond tobacco control.

As a new international legally binding treaty, the WHO FCTC and its Protocol opened a new area in global health governance.^2^ This treaty has accelerated the debate around and expectations regarding the role of legal regimes at an international level in global health. Nikogosian and Kickbusch outline some of the implications of this treaty, including that it has prompted a stronger role for national legislation for health – and indeed that achieving the health benefits of international law in general requires translation into domestic law.^2^

Some commentators have argued that international treaties have the potential to result in great benefit for global health, but also harm, given they are accompanied by, as described by Hoffman et al, “high costs, risks of harm, and trade-offs.”^3^ They propose four criteria to assess proposals for new global health treaties. First, the problem addressed by the (proposed) new treaty should have a significant transnational dimension, involving multiple countries, transcending national borders, and transferring risks of harm or benefit across countries. Second, the problem should justify the coercive nature of the proposed solution. Third, there should be a reasonable chance of achieving the intended benefits: the treaty should incentivise decision-makers, institutionalise accountability mechanisms, and encourage advocates. Fourth, a treaty should be the best available option, considering costs and benefits, if compared with alternative mechanisms. Not many proposals would pass the test of these four criteria, if applied strictly. However, in a later paper, Hoffman et al conclude that the problem of antimicrobial resistance (AMR) passes the test and deserves a new treaty.^3,4^

We would argue that, if judged by the standards of Hoffman et al, even the problem of AMR may not deserve a new treaty. Existing international treaties could achieve the same result, if used creatively by civil society, and if courts are willing to acknowledge that the necessity of participation by most countries cannot justify the paralysis of individual countries. That is what happened in the Netherlands in a court case about a very different issue that comes with similar challenges, and is also an issue of critical importance to global health,^5^ namely climate change. In 2015, the District Court of The Hague ordered the Dutch government to cut greenhouse gas emissions by at least 25% within five years (by 2020), relative to 1990 levels. The judges involved in the case ruled that cutting emissions by a lesser 14%-17% by 2020 – as per government plans – was unlawful, given the threat posed by climate change.^6^ The case was filed by 886 Dutch citizens organised by Urgenda, an environmental non-profit organisation. The plaintiffs accused the Dutch government of negligence, through breaching the 2°C maximum target for global warming.^7,8^ The ruling was based on a combination of Dutch civil law (the duty of care) and existing human rights and climate change treaties. The Court followed the plaintiff’s reasoning regarding the complexity of the issue and the multiple actors involved, and decided that the multitude of duty-bearers was not an excuse for each of them individually to refrain from action. As described in the verdict: “The State should not hide behind the argument that the solution to the global climate change problem does not depend solely on Dutch efforts. Any reduction of emissions contributes to the prevention of dangerous climate change and as a developed country the Netherlands should take the lead in this.” Some commentators have suggested that such reasoning may be used in the courts of other countries, with speculation that the Dutch judgement could inspire a global civil movement to address climate change.^7^ Indeed, similar cases addressing climate change are now being prepared in elsewhere, and some governments are losing climate lawsuits, including most recently in South Africa after the high court ruled against government plans for a coal-fired power station.^9^ What is relevant for this comment, however, is that the same logic could also be applied to AMR: every government that does not adopt appropriate measures contributes to the problem and contributes to a violation of the right to health; any reduction of inappropriate use of antibiotics contributes to the prevention of AMR, and no government can legitimately hide behind the fact that success in preventing AMR depends on other states too. If so, a new treaty on AMR may not be needed after all.

To be clear, we are not trying to make the point that new treaties are redundant. On the contrary, we agree with Nikogosian and Kickbusch: new international treaties to address urgent health problems can be very useful, even when alternative mechanisms may work too. The point we are trying to make is that law is indeed an ‘undervalued tool’ for addressing issues affecting health and wellbeing, with great potential as described by Nikogosian and Kickbusch,^2^ and that it is ‘underused’ in two ways: not only is the possibility of new international treaties ‘underused,’ but also existing international treaties are ‘underused’ in national courts. The Dutch court ruling provides an example of a civil-society driven domestic strategy for achieving governmental regulatory change. Of course, such a legal strategy will not be replicable across all country settings. Some countries do not have legal systems within which such a case could be brought against government. The strategy also requires a mobilised civil society, a well-functioning, democratic government and an effective judicial system. However, cases using the same logic in a handful of countries could encourage these countries to advocate for stronger and clearer new treaties, to share the burden fairly.

## Conclusion

The Dutch court ruling about climate change illustrates how international law is becoming an increasingly important instrument of global governance, opening the door for a new kind of innovation in global health governance,^10^ which can be used by civil society to hold governments accountable, not only by states holding each other accountable. The possibility of using similar strategies to address other global health issues adds a new dimension to the use of law in global health governance, as described by Nikogosian and Kickbusch.

## Ethical issues

Not applicable.

## Competing interests

Authors declare that they have no competing interests.

## Authors’ contributions

Both authors both contributed conceptually to the development of the article and to drafting the manuscript.
